# Correlation Between Teamwork and Patient Safety in a Tertiary Hospital in Cyprus

**DOI:** 10.7759/cureus.19244

**Published:** 2021-11-04

**Authors:** Mary Kyriacou Georgiou, Anastasios Merkouris, Maria Hadjibalassi, Pavlos Sarafis, Theodoros Kyprianou

**Affiliations:** 1 Quality Assurance Department, Nicosia General Hospital, Nicosia, CYP; 2 School of Health Sciences, Cyprus University of Technology, Limassol, CYP; 3 Nursing Department, Cyprus University of Technology, Limassol, CYP; 4 Nursing Department, University of Thessaly, Lamia, GRC; 5 Medicine, University of Nicosia, Nicosia, CYP; 6 Respiratory and Intensive Care Medicine, King’s College Hospital NHS Trust, London, GBR

**Keywords:** deepening our understanding of quality improvement in europe, safety attitudes questionnaire, hospitals, health professionals, teamwork, safety climate

## Abstract

Background

Over time, the multidimensional nature of the safety culture in the healthcare field has led to great efforts to improve quality and create tools aiming at enhancing safety. In particular, emphasis has been placed on teamwork and the safety climate. There is a strong relationship between these two complex elements, which interact to improve the safety climate and reduce patient-safety issues. In this study, “teamwork” includes the perceptions of the health professionals collaborating within a health team to provide safe patient care, and “safety climate” refers to the professional commitment to patient safety.

Objective

This article assesses health professionals’ perceptions of both patient-safety issues and teamwork in their hospital work environment after the development and implementation of a comprehensive quality-assurance system.

Methods

This descriptive correlation study is based on anonymous and self-completed questionnaires obtained after the development and implementation of a comprehensive quality assurance system in the wards and departments of Nicosia General Hospital. The research sample consisted of the health professionals who participated in the working groups that implemented the quality assurance system. We used the questionnaire’s sociodemographic data and the Safety Attitudes Questionnaire (SAQ) developed in the Deepening our Understanding of Quality Improvement in Europe program, focusing on two factors: Teamwork and the safety climate.

Results

While teamwork received a positive score (>75%), the same did not occur for the safety climate (68.60%). Women typically rated the safety climate more positively than men, who mostly gave negative ratings (p = 0.005). There was a statistically significant difference (p = 0.011) in the scores between participants aged 24-44 and those aged 45-54, with the latter reporting higher teamwork scores. The participants’ educational levels also played important roles in their responses, with university graduates (BSc) providing more positive teamwork scores than those with a master’s degree (p = 0.018).

Conclusions

Our research revealed that the health professionals of Nicosia General Hospital perceived the teamwork climate as positive, in contrast to the safety climate. The results highlight the need not only to intervene in all the areas covered by the SAQ to improve the safety climate but also to keep encouraging teamwork to obtain better results.

## Introduction

Safety culture in the healthcare field is a complex and enduring feature. It reflects fundamental values, rules, assumptions, and expectations that concern not only the health sector but also the whole of society. Over time, the multidimensional nature of the safety culture in the field of health care has led to great efforts to improve its quality and to create tools aimed at bettering safety. At the same time, research has focused particular emphasis on teamwork and the safety climate, which are two complex elements that interact strongly to improve the safety climate and reduce patient-safety issues [1]. Here, “teamwork” includes health professionals’ perceptions when collaborating within the healthcare team to provide safe patient care, and “safety climate” refers to the professional commitment to patient safety [1].

The key ingredients for healthcare organizations that want to maintain or improve safety include staff who feel psychologically safe, appreciate and respect diversity, and share an exciting vision; good leadership at all levels; a sense of teamwork; and openness and support for learning [2]. Health professionals must feel that they are working in an inclusive environment to be able to work their best. Indeed, psychological security works at the team level - not the individual level - with each person knowing that they will be treated fairly and compassionately by the team if things go wrong [3]. This means that staff does not feel the need to behave defensively to protect themselves, instead of opening up to the team so that learning can take place [4]. Group psychological security is characterized by an atmosphere of participation, trust, and respect, where people feel able to prosper as individuals. Accordingly, valuing diversity plays a critical role, and it is vital to recognize how beneficial age, gender, nationality, strength, or diversity of thinking is for teamwork, communication, and effective performance. If utilized properly, these differences encourage learning and creativity. When the management leading a team strengthens the voice of even the least-powerful members, this enhances the safety climate [3-4]. Conversely, deficit-based work that undermines, humiliates, or worsens discrimination against those who are different leads to fear and reduces group psychological security, as well as learning, in the workplace [5].

Before good leadership can be implemented, there must be a shared vision of what a team wants to achieve, including a good understanding of why something is done and what is wanted to have a successful system; the vision must be explicit and be accepted by everyone. Organizations that emphasize the importance of long-term thinking and strategy, and that have high team ambitions, encourage pride and positivity in the workplace. Good leadership creates psychological security and encourages team members to pay attention to each other, develop mutual understanding, and empathize with and support each other. Groups with these traits are also extremely innovative [5-6]. The way leadership is exercised in an organization is thus critical to its success, and clinical leadership is particularly important for safety. In addition, team members must learn to protect each other and encourage their commitment to the requirements of the organization they work for. Also, if they have a clear role in the team, they are less likely to be exhausted and more likely to operate with safety [3-6]. To develop a learning culture, though, a health team must focus on what needs to be changed, not on punitive actions. An organization that identifies, contains, and recovers from errors as quickly as possible is, in turn, mindful of possibilities for learning and continuous improvement [3-6].

A search of relevant literature reveals that this is the first study from the Republic of Cyprus that attempts to evaluate the views of health professionals about quality following a quality-assurance intervention. The present research was based on the conceptual model of the Deepening our Understanding of Quality Improvement in Europe (DUQuE) study, which was developed with the participation of researchers from different cognitive backgrounds and specialties in many European countries [7-10]. Specifically, this research was carried out at Nicosia General Hospital, Cyprus’s largest tertiary public hospital, with 500 beds. We expect this evaluation of health professionals’ views regarding patient safety and teamwork to contribute to the development of clinical practice, the improvement of working conditions, and the assurance and improvement of healthcare quality.

## Materials and methods

Study design and setting

This cross-sectional study is based on anonymous and self-completed questionnaires obtained after the development and implementation of a comprehensive quality-assurance program in the wards and departments of Nicosia General Hospital.

Participants

The research sample consisted of the health professionals who participated in the working groups that implemented the quality assurance system.

The sample-selection criteria were as follows: 1. Personal desire to participate in the research; 2. Staff who participated in the working groups that created the quality system; 3. Medical and nursing staff from all departments and specialties; 4. Staff who were trained in quality-system management in the past.

The participants were informed that they could withdraw from the study at any time without any consequence to their working life, as their participation was voluntary.

We selected a total of 250 people to complete the questionnaire. In total, 174 (70%) responded, which adequately represents the team members who participated in the working groups that implemented the quality assurance system. We determined the sample size to achieve the desired level of accuracy of the results, incur reasonable financial costs, and fit within available time margins. The specific sampling method was chosen to prevent problems in gathering the sample. The confidence interval, or margin of error, in the questionnaire was 0.05.

Evaluation tool and data collection

The questionnaire we used consisted of two parts: The first part asked about the respondent’s sociodemographic data and outlined the characteristics of the assignment. The second part included the Safety Attitudes Questionnaire (SAQ), which was developed by Sexton et al. (2006) [7], and used in the DUQuE program. The questions concern the following six factors: Teamwork (6 questions), safety climate (7 questions), Work Satisfaction (5 questions), Stress Recognition (4 questions), Management Perception (4 questions), and Working Conditions (4 questions). From this scale, which was translated and validated in Greece and Cyprus [8], we selected two factors: Teamwork and Safety Climate. We chose these factors after a thorough literature review revealed that teamwork is recognized as an important component in the organization of services that provide high-quality care to patients and, in particular, in creating a climate of patient safety. As indicated above, six questions address the teamwork factor, one of which has a reverse rating, and seven questions address the safety-climate factor, of which one also has a reverse rating. All items were scored on a Likert scale ranging from 1-5 (strongly disagree to strongly agree). We converted the answers to a scale of 0-100 as follows: 1, 0%; 2, 25%; 3, 50%; 4, 75%; and 5, 100%. We considered results greater than 75% to be positive. We also considered responses between 65% and 75% as a strong tendency toward a positive attitude, 50% to 65% as a tendency toward a positive attitude, and below 50% as a negative attitude. For the two negatively formulated questions, the corresponding scores were 5, 0%; 4, 25%; 3, 50%; 2, 75%; and 1, 100%.

Ethical considerations

In the preparation of this paper, we requested and secured approval from the Commissioner of Personal Data Protection, the Cyprus National Bioethics Committee, and the Research Promotion Committee of the Ministry of Health of Cyprus. Each subject’s participation in this research was entirely voluntary, and they were asked to provide their written consent to participate before completing the questionnaire. The participants were informed in writing about the purpose of the study, and they were asked not to fill in any personal identification data since their answers were to be completely anonymous. The responses were used solely for the purpose of this research and were reviewed only by the research team. All the data collected were stored by the researchers with absolute confidentiality and security.

Statistical analysis

For the analysis and processing of statistical data, we coded all the answers and entered them into the Statistical Package for Social Sciences, version 21. For the analysis of demographic data, we used a descriptive statistic that provided the average, standard deviation, and minimum and maximum values for the quantitative variables and the frequencies and percentages for the qualitative variables, together with 95% confidence intervals. We also performed reliability analyses of all the scales using Cronbach’s Alpha coefficient. We obtained inductive statistics using nonparametric tests, special correlation coefficients, and an independent t-test to investigate the correlation between knowledge, attitudes, and compliance between different occupational groups.

## Results

The vast majority of the sample consisted of women, over the age of 35, married, university graduates (30% held a master’s degree), and who had attended a transitional nursing-education program. The average length of service was 12.95 ± 12.55 years.

Safety-attitude questionnaire

Table 1 and Table 2 show the response rates to each question for the two SAQ factors. For the teamwork factor, the three questions answered most positively, included the item concerning the staff’s ease in asking about something they did not understand (86.2%); the item “Nurse input is well received in this clinical area” (90.5%); and the item “I have the support I need from other professionals in patient care” (81.8%).

**Table 1:** Response to questions about teamwork

	Strongly Disagree	Slightly Disagree	Neutral	Slightly Agree	Strongly Agree	Mean Score	Standard Deviation
Nurse input is well received in this clinical area	1.2%	1.8%	6.5%	47.9%	42.6%	4.29	0.77
In this clinical area, it is very difficult to speak up if I perceive a problem with patient care	39.6%	30.8%	7.7%	17.2%	4.7%	2.17	1.25
Health-care workers here work together as a well-coordinated team	0.0%	8.8%	8.8%	50.3%	32.2%	4.06	0.87
Disagreements in this clinical area are resolved appropriately (i.e. not who is right but what is best for the patient)	3.5%	9.4%	15.2%	46.8%	25.1%	3.81	1.03
It is easy for personnel in this clinical area to ask questions when there is something that they do not understand	4.7%	3.6%	4.1%	27.2%	60.4%	4.35	1.05
I have the support I need from other personnel to care for patients	0.0%	9.4%	8.8%	45.9%	35.9%	4.08	0.91

**Table 2:** Response to questions about safety climate

	Strongly Disagree	Slightly Disagree	Neutral	Slightly Agree	Strongly Agree	Mean Score	Standard Deviation
I would feel safe being treated here as a patient	3.0%	6.5%	14.9%	41.7%	33.9%	3.97	1.01
I am encouraged by my colleagues to report any patient safety concerns I may have	0.6%	6.1%	14.6%	47.0%	31.7%	4.03	0.88
The culture in this clinical area makes it easy to learn from the errors of others	2.4%	9.0%	21.6%	46.7%	20.4%	3.74	0.96
I receive appropriate feedback about my performance	4.2%	13.2%	23.4%	39.5%	19.8%	3.57	1.08
Medical errors are handled appropriately in this clinical area	6.1%	9.1%	24.4%	37.8%	22.6%	3.62	1.12
I know the proper channels to direct questions regarding patient safety in this clinical area	1.8%	8.5%	14.0%	49.4%	26.2%	3.90	0.95
In this clinical area, it is difficult to discuss errors	23.0%	32.7%	14.5%	21.8%	7.9%	2.59	1.27

For the safety-climate factor, there were also three most-positive responses. The first concerned the item “I am encouraged by my colleagues to report any patient safety concerns” (78.7%). The health professionals also answered that “I would feel safe being treated here as a patient” and that “I know the proper channels to direct questions regarding patient safety in this clinical area,” each at a rate of 75.6%.

The lowest scores for both factors were for the items “In this clinical area it is difficult to discuss errors” and “In this clinical area it is very difficult to speak up if I perceive a problem with patient care.” The next lowest were the item “I receive appropriate feedback about my performance,” at 17.4%, and the item “Disagreements in my clinical space are resolved appropriately (i.e. not who is right, but what is best for the patient),” at 12.9%.

Table 3 shows the overall factor scoring for teamwork and safety climate based on the transformations described in the data collection subsection. The total score for the teamwork factor was 76.68%, which - at a rate higher than 75% - we consider positive. However, the safety-climate factor had a total score of 68.60%, which we consider to be negative. Additionally, for teamwork, Cronbach’s Alpha coefficient was 0.650, while for safety climate, it was 0.789. These values show that the responses given to the safety-climate questions had higher internal consistency than the responses given to the teamwork questions.

**Table 3:** Overall scores of the two SAQ factors, teamwork and safety climate (%) SAQ: Safety Attitudes Questionnaire

	N	Minimum Score	Maximum Score	Mean Score	Std. Deviation
Teamwork	171	33%	100%	76.68%	14.96%
Safety Climate	169	18%	100%	68.60%	17.32%

The effects of sociodemographics on the teamwork and safety climate responses

Women scored higher than men with regards to safety climate (p=0.005) and marginally higher (p=0.061) than men with regards to teamwork (Table 4). Women demonstrated a positive perception of teamwork and a tendency towards a positive perception of safety climate, whereas men's perception shows a strong tendency to be positive for teamwork and a tendency toward positive for safety climate.

**Table 4:** Predictors affecting the SAQ factors, teamwork and safety climate SAQ: Safety Attitudes Questionnaire

		N	Teamwork Mean score	Std. Deviation	p-value	Safety Climate Mean score	Std. Deviation	p-value
Gender	Male	44	73.01%	16.18%	0.061	60.80%	20.72%	0.005
	Female	118	78.03%	14.62%		70.97%	15.24%	
Work Experiences	≤ 5.00	43	80.43%	12.61%	0.024	69.52%	17.48%	0.16
	5.01–20.00	24	70.49%	14.48%		61.01%	17.20%	
	20.01+	37	73.99%	17.52%		63.69%	20.39%	
Age	25–44	39	71.11%	13.18%	0.006	63.55%	18.74%	0.17
	45–54	74	80.05%	13.87%		69.86%	16.51%	
	55+	45	74.12%	16.84%		68.83%	17.48%	
Education	Technological institute	12	73.96	12.45%	0.016	67.26	11.17%	0.085
	University (BSc)	91	79.66	13.59%		70.87	15.35%	
	Postgraduate studies (Master Degree)	48	71.96	16.18%		63.39	19.95%	
	Doctoral studies	13	72.12	16.52%		65.11	20.05%	

Additionally, people with ≤ 5.00 years of service gave the highest scores for the teamwork factor as compared to people with 5.01-20.00 years of experience (p = 0.024).

The response of age groups with regards to teamwork is positive for age group 25-44 (80.05%) and presents a strong tendency for positive perception for the age groups 25-44 (71.1%) and 55+ (74.12%).

There was a statistically significant difference (F (2,155) = 5.332 p=0.006) at the 95% confidence level, between participants aged 25-44 and those aged 45-54, with the latter reporting higher teamwork in their clinical areas. All three age groups (25-44, 45-54, and 55+) reported positively on teamwork (>75%; Table 4) but not on safety climate, for which no age group gave a positive score. Nevertheless, as regards the perception of safety climate, there is a tendency for a positive response for all examined age groups as the mean scores lie between 60% and 70%. The three group responses do not present any statistically significant difference (F (12,155) = 1.804, p=0.168).

As for the relationship between educational level and response on teamwork (Table 4), university graduates (BSc) respond positively(79.6%), whereas the rest of the groups present a strong tendency toward a positive response with scores between 71% and 75%. The multiple group comparison indicates a statistically significant difference between the group of university graduates and the group of participants with a postgraduate master’s degree (F (3.160) = 3.53, p=0.016) at the 95% confidence level, in favor of the former.

The analysis of response on safety climate in relation to educational level indicates that participants at all education levels present a tendency towards a positive response with scores between 65%-71%. The response of the studied education level groups does not present any statistically significant difference at 95% confidence level (F (3.160) = 2.243, p=0.085).

We further investigated the correlation between the perception of participants on teamwork improvement and the corresponding safety climate improvement as a result of the implementation of a quality system. By means of a Pearson correlation test on the participants' scores for the two parameters (Table 4), we found a strong positive correlation r (10) = 0.88, p<0.001) at the 95% confidence level.

## Discussion

This research took place after the development and implementation of a comprehensive quality assurance program at Nicosia General Hospital. The project was implemented by working groups consisting of health professionals (mainly physicians and nurses) who had been previously trained in quality systems. A search of the relevant literature revealed that this is the first research from the Republic of Cyprus that attempts to evaluate health professionals’ views on quality following an intervention to ensure it. The present research was based on the conceptual model of the DUQuE study, which was developed by researchers with different cognitive backgrounds and specialties to determine the effectiveness of quality-improvement systems in European hospitals [7-10]. As Nicosia General Hospital implemented its own comprehensive quality assurance program, we considered this tool to be the most suitable one for this study.

This study used the SAQ because it is a questionnaire specifically designed for the assessment of perceptions concerning the safety climate in hospitals and among various health professionals, both individually and as a group. As mentioned above, we chose these factors because of the importance of teamwork in improving and ensuring the safety climate. The evaluation showed that while the teamwork factor received a positive rating, given that its overall score was >75%, the same did not occur for the safety-climate factor. Two other surveys carried out within Cypriot public hospitals, in one of which the participants were exclusively nurses working in an ICU, yielded a positive overall teamwork score; in contrast, the scores were quite low in another survey of obstetric staff [11-14]. Both the teamwork climate and job satisfaction were high in the ICU, possibly due to the way the department operates and to the possibly greater autonomy of the nursing staff. It is important to emphasize that the scores obtained in the present research were obtained from all the medical and nursing staff, in contrast to the research from the ICU, which involved exclusively nursing staff [15].

Further analysis of the medical and nursing staff’s responses revealed no statistically significant differences in the two factors’ average overall scores, which remained positive for teamwork. Potentially, the health professionals involved had developed a close-knit team before, or in the process of, creating the hospital’s quality-assurance program, which carried over into their daily work interactions. Cronbach’s alpha coefficients for the two SAQ factors also differed from the acceptable values of >0.70. In particular, the value for the teamwork scale was 0.65.

As other surveys from around the world also have revealed, the internal consistency of the teamwork perception rate is relatively low as opposed to the safety-climate rate [13-16].

Factors influencing safety climate and teamwork

Analysis of present study survey data as regards the perception of health professionals on teamwork and safety climate after the implementation of a quality system indicated either a positive perception or a tendency toward positive perception. No negative perception was recorded, and this is not a surprise, as the implementation of a quality system is expected to improve processes and procedures, enhancing the teamwork approach and safety perception. In general, the perception of improvement in teamwork after the implementation of a quality system is stronger than the safety climate. Moreover, the general perception of improvement is stronger among women as compared to men.

In particular, a positive perception of teamwork is recorded among women, professionals with limited experience (≤ 5 years), as well as the middle-aged (45-44), who have a university BSc-level academic background. A strong tendency for a positive perception is recorded among the rest of the studied groups. On the other hand, no group demonstrated a positive perception of improvement in safety climate after the implementation of a quality system. However, the groups of women, people with work experience less than five years as well as people over 45 years of age, at all studied levels of education except for the level of master’s degree, exhibited a stronger tendency towards a positive perception of safety climate improvement than the rest of the groups.

Furthermore, the strong positive correlation between the perception of participants on teamwork improvement and the corresponding on safety climate improvement, as a result of the implementation of the quality system, supports the hypothesis that the promotion of teamwork through the implementation of a quality system results in the enhanced perception of safety among personnel establishing a stronger safety climate.

In Taiwan, for example, the Ministry of Health implemented the Taiwan Clinical Performance Indicator System and the Health Quality Improvement Campaign, which both resulted in improved teamwork (from 50.2% in 2009 to 58.6% in 2016 in a sample of 23,780 health professionals) and a safety-climate change (from 46% in 2009 to 54.3% in 2016 in a sample of 85,542 health professionals) [17]. Additionally, from Taiwan [18], researchers found that physicians and nurses who participated in quality-control programs gave more positive and statistically significant scores for the factors safety climate and teamwork climate. Thus, the development and implementation of quality assurance programs, such as the one implemented at Nicosia General Hospital, seem to improve health professionals’ views of both teamwork and safety climate, which are dimensions of service quality. It would therefore be interesting to re-evaluate these two factors in a future investigation at Nicosia General Hospital to obtain further remarks from those involved in the program’s implementation as well as from the rest of the staff.

In the present study, we found that safety climate was related to gender (men had the lowest scores) and marital status (married with or without children had the highest score). In similar studies from the rest of the world, physicians had a more positive view of teamwork but not of safety climate [16-20], with the exception of the research by Nguyen et al. from Italy [21]. In another study, physicians scored the safety climate higher, but the qualitative data from that study revealed a number of issues that the hospital staff felt impacted negatively on patient safety [22]. Some of these concerns were the shortage of staff, the need to minimize barriers to improving patient safety, and the need for more patient-focused care. The study also showed that cultivating a safe culture for error reporting and improving opportunities for training and education can have positive impacts on patient safety.

Teamwork factor

For the teamwork factor, the most positive answers were to the two items “It is easy for personnel in this clinical area to ask questions when there is something that they do not understand” and “Nurse input is well received in this clinical area.” These items also had the highest scores in other surveys [1,11-12,22], which indicates that teamwork in different countries is defined in terms of the possibility of collaborative staff feedback and nurse performance. These two parameters, therefore, indicate that it is crucial to maintain a strong teamwork-based climate at the management level.

It is worth mentioning that more than half of the health professionals had formal administrative roles at the department level while only one-fifth were at the hospital level and not paid for the time they devoted to administrative work. This may explain the positive results at the teamwork level, as the sample consisted mainly of people in administrative positions.

Safety-climate factor

For the safety-climate factor, the two most positively answered items were “I am encouraged by my colleagues to report any patient safety concerns” and “I would feel safe being treated here as a patient.” These two items were the most prevalent among the various high scores in other surveys as well [1,11-12,21-22]. It seems then that a hospital’s safety climate is determined by open discussions about patient safety issues and a sense of security.

Limitations

The present research has some limitations. The first involves the study’s focus on a single hospital, which was the first public hospital in Cyprus to implement a quality assurance system. This fact not only limited the sample employed in this investigation, but also it means that the results cannot be compared with other public hospitals in Cyprus. The second limitation concerns the point at which safety climate, teamwork, and the role of staff in quality processes and activities were assessed. In the future, a larger study like this one could provide important information on the changing culture of quality and safety.

## Conclusions

From these results and comparisons with corresponding data from the international literature, some conclusions emerge that can form the basis for management-level changes to ensure the sustainability and improvement of health-quality-assurance programs. Notably, the participating staff perceived teamwork more positively than they did the safety climate. However, the implementation of a comprehensive quality system establishes a positive perception for both teamwork and safety climate, as they are strongly correlated. The statistically significant results of this survey focused mainly on the predictive factors for teamwork [marital status (married with children), educational level (university graduate), and seniority (lesser)]. Also, the development of the quality assurance program, which facilitated cooperation among the staff (medical and nursing), most likely demonstrated the benefit of teamwork. Additional data on the overall teamwork score before the program’s implementation might have revealed differences that could have substantiated this hypothesis. As for the safety climate, gender had a statistically significant role, with men providing more negative scores, while marital status (participants married with or without children) produced more positive scores. The implementation of a comprehensive quality system establishes a positive perception for both teamwork and safety climate as they are strongly correlated.

Ultimately, both safety climate and teamwork must be evaluated systematically so that targeted actions can be taken to resolve any issues that may arise and to ensure a safe climate. While this is primarily for the benefit of patients, it also benefits health professionals and encourages compliance with quality assurance or certification interventions.
